# Acute Pancreatitis in Pancreas Divisum Secondary to an Impacted Stone in the Minor Papilla

**DOI:** 10.7759/cureus.5481

**Published:** 2019-08-25

**Authors:** Rehan Farooqi, Carol Burke, Prabhleen Chahal, Faris El-Khider, Umar Zahid

**Affiliations:** 1 Internal Medicine, Medstar Union Memorial Hospital, Baltimore, USA; 2 Gastroenterology, Cleveland Clinic, Cleveland, USA; 3 Internal Medicine, Johns Hopkins Bayview Medical Center, Baltimore, USA

**Keywords:** pancreas divisum, acute recurrent pancreatitis, minor papilla, pancreatic duct stone, endoscopic ultrasound, cholelithiasis, bile ducts, ercp, major papilla

## Abstract

Pancreas divisum is reported to occur in up to 14% of the population. The majority of patients with this congenital anomaly remain asymptomatic. Pancreas divisum can be associated with recurrent pancreatitis due to inadequate drainage of pancreatic secretions through the dorsal pancreatic duct and the minor papilla. We present a patient with a six-month history of recurrent acute pancreatitis due to an impacted pancreatic duct stone in the minor papilla and an unrecognized pancreas divisum. This situation has only been reported in two other cases in the literature.

## Introduction

Pancreas divisum (PD) is a common congenital pancreatic duct anomaly occurring in 4%-14% of the population [1]. It results from a failure of fusion between the dorsal and ventral pancreatic ducts during the seventh week of embryogenesis [1]. Three variants of PD have been described: type 1 is the total failure of fusion of the ventral and dorsal pancreatic ducts; type 2 is the complete absence of the ventral duct; and type 3, incomplete divisum, is where a small communication is present between the ventral and dorsal pancreatic ducts [2]. In approximately 5% of patients, the anomaly is associated with recurrent pancreatitis because of the inadequate drainage of pancreatic secretions through the dorsal duct via the minor papilla [2]. In PD, the ventral duct drains the inferior and posterior parts of the head of the pancreas through the major papilla. The dorsal duct drains the superior and anterior parts of the head as well as the body and tail of the pancreas through the minor papilla [3]. We present a patient with recurrent acute biliary pancreatitis in the setting of PD with a dorsal pancreatic duct stone impacted in the minor papilla unrecognized until endoscopic ultrasound (EUS) was performed.

## Case presentation

A 65-year-old male with essential hypertension and a history of heavy alcohol use presented to the hospital with dull, unremitting epigastric pain radiating to the back for the past three weeks. He had associated nausea, early satiety, and anorexia. He had no fever, chills, emesis, jaundice, nor changes in bowel movements. On examination, his blood pressure was 148/83 mmHg, heart rate was 83 beats per minute, and he was afebrile. No scleral icterus was noted. He had a soft abdomen with mild epigastric tenderness, no palpable organomegaly, and bowel sounds were present. The remainder of his physical examination was noncontributory. Pertinent laboratory tests at that time included a leukocyte count of 4,700 K/uL, hemoglobin 13.2 g/dl, hematocrit 39%, creatinine 0.72 mg/dl, blood urea nitrogen 4 mg/dl, lipase 250 U/L (range 16-61 U/L), alkaline phosphatase 168 U/L (range 32-117 U/L), with normal bilirubin and transaminase levels. Computed tomography (CT) of the abdomen reported a calcified stone in the pancreatic duct with a dilated duct in the body and tail of the pancreas and an additional stone in the duct at the proximal body of the pancreas. An abdominal ultrasound revealed cholelithiasis. The patient underwent endoscopic retrograde cholangiopancreatography (ERCP) with biliary sphincterotomy but pancreatic duct cannulation was unsuccessful. Laparoscopic cholecystectomy was performed for presumed gallstone pancreatitis and the patient was discharged home.

**Figure 1:**
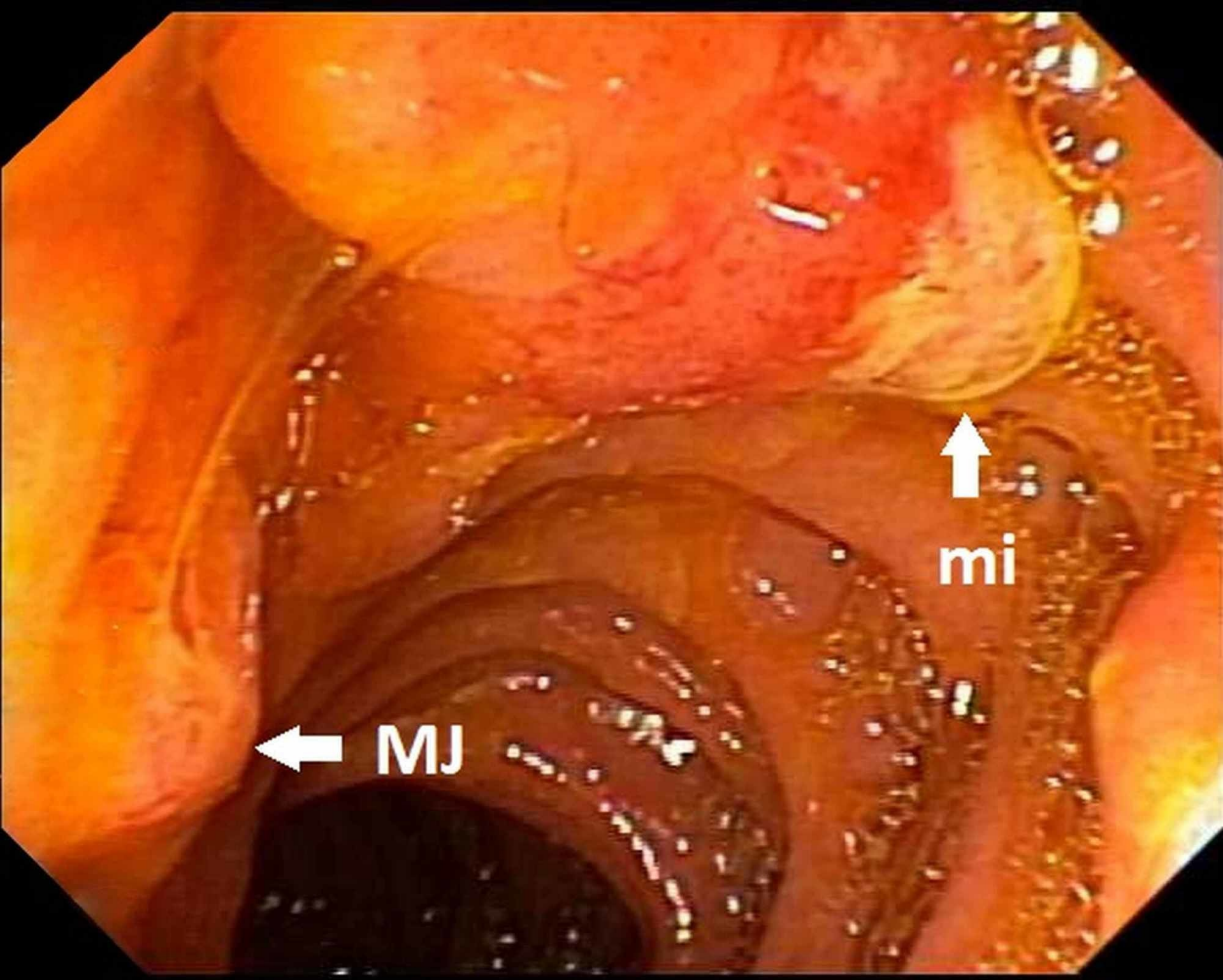
Bulging minor papilla (mi) before sphincterotomy and normal-appearing major papilla (MJ)

Abdominal pain recurred and five weeks later, the patient was readmitted with acute pancreatitis. Magnetic resonance cholangiopancreatography (MRCP) demonstrated a 5-mm filling defect in the mid pancreatic duct. An ERCP was repeated but attempts at pancreatic duct cannulation were unsuccessful. Pancreaticoduodenectomy was recommended to the patient, and he was transferred to Cleveland Clinic for a second opinion. Endoscopic ultrasound (EUS) was performed, which demonstrated Type 1 PD, an impacted stone at the minor papilla with dorsal duct dilation, and changes of chronic pancreatitis. ERCP was performed next and the minor papilla was found to be bulging (Figure 1).

**Figure 2:**
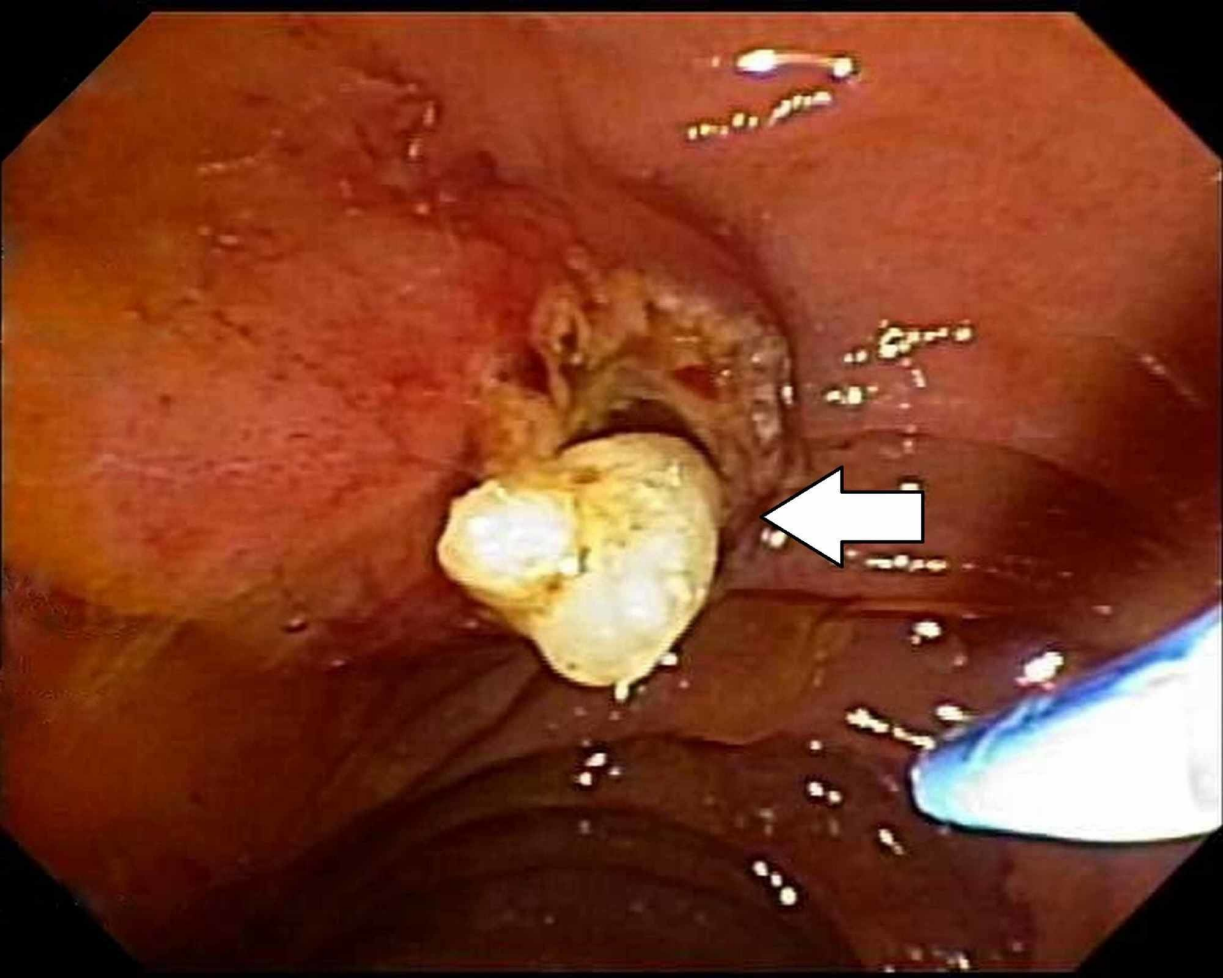
The arrow depicts minor papilla stone extraction after sphincterotomy

Dorsal pancreatic sphincterotomy was performed and prompt egress of a single 4-mm stone occurred (Figure 2).

A temporary stent was placed into the dorsal pancreatic duct. The patient reported the resolution of his abdominal pain after the procedure. There were no procedure-related complications.

## Discussion

Over 95% of patients with PD are asymptomatic while the remaining 5% develop symptoms of acute pancreatitis [4]. In the absence of ductal stones, pancreatitis develops in PD because of increased ductal pressure secondary to insufficient drainage of pancreatic secretions through the minor papilla [4]. Our patient, unfortunately, was in the 5% category who developed recurrent symptoms of acute pancreatitis. The reason as to why only a select few patients develop symptoms is not clear. The diagnosis of PD is often delayed due to the inadequate sensitivity of conventional radiographic cross-sectional imaging like magnetic resonance imaging (MRI) of the abdomen. The reported accuracy of EUS, MRCP, and multi-detector computed tomography (MDCT) to detect PD is variable and appears to relate to imaging protocols, the expertise of radiologists interpreting the study, and the skill of the endoscopist. In one study, the sensitivity of EUS was 86.7% higher than the sensitivities of MDCT (15.5%) and MRCP (60%) [5]. In a detailed systematic review and meta-analysis, MRCP, secretin-enhanced MRCP, and EUS were compared to address the diagnostic accuracies in the detection of pancreas divisum. It was concluded that EUS was more sensitive at 85% when compared to MRCP (59%) and secretin-enhanced MRCP (83%). All three imaging modalities had specificities above 97% [6].

This is a unique case with many important clinical points to consider. This is one of three reported cases of acute pancreatitis in a patient with PD due to an impacted stone at the minor papilla [7-9]. The two previously published cases were in 1999 from Switzerland and in 2016 from Japan [8-9]. This challenging case is interesting, as our patient experienced recurrent acute pancreatitis due to an impacted stone in the minor papilla in the setting of PD, which went unrecognized until EUS demonstration of the congenital anomaly. His symptoms resolved after minor papilla pancreatic sphincterotomy with the retrieval of the stone at ERCP. He was symptom-free at the six-month follow-up post-discharge.

## Conclusions

The two most common causes of acute pancreatitis in the United States are alcohol abuse and cholelithiasis. Other causes include trauma, medications, infections, hypertriglyceridemia, pancreas divisum, and hereditary and auto-immune conditions. As internist and specialist, it is important to consider different etiologies of acute pancreatitis and ordering tests cost-effectively to narrow the differential. Our patient quit alcohol several years ago and was status post-cholecystectomy for presumed gallstone pancreatitis prior to presenting to Cleveland Clinic. However, he continued to have recurrent symptoms of acute pancreatitis. The diagnosis of pancreas divisum must be considered for which EUS would be the diagnostic modality of choice, based on its sensitivity and specificity.
